# How has the management of acute coronary syndrome changed in the Russian Federation during the last 10 years?

**DOI:** 10.1016/j.healthpol.2017.09.018

**Published:** 2017-12

**Authors:** Anna Kontsevaya, Tamara Sabgaida, Alla Ivanova, David A. Leon, Martin McKee

**Affiliations:** a Department of Non-communicable Disease Epidemiology, National Research Center for Preventive Medicine, Moscow, Russian Federation; b Department of Statistics of Population Health, Federal Research Institute for Health Organization and Informatics, Moscow, Russian Federation; c Department of Non-communicable Disease Epidemiology, London School of Hygiene and Tropical Medicine, London, UK; d Department of Health Services Research and Policy, London School of Hygiene and Tropical Medicine, London, UK

**Keywords:** Acute coronary syndrome, Russian Federation, Access to medical care, Percutaneous cardiovascular interventions

## Abstract

**Methods:**

We report trends and patterns of percutaneous cardiovascular interventions (PCI) by region for 2005–2009, with more detailed data on management of myocardial infarctions in 2009–2103, relating them to regional economic development and changes in mortality from myocardial infarction.

**Results:**

PCIs per 100,000 population increased from 8.7 in 2005–71.3 in 2013, with considerable regional variation. In 2013 the highest rates were in the wealthiest regions, although not in some remote regions dependent on oil and mineral extraction. Between 2009 and 2013 rates of thrombolysis in those with acute myocardial infarctions potentially eligible for treatment remained broadly similar at about 28% but rates of primary revascularisation with stenting rose rapidly, from 6.5% to 23.7%. In-hospital mortality from myocardial infarction since 2009 has declined most in regions achieving highest rates of primary revascularisation.

**Conclusions:**

The sustained investment in advanced cardiovascular technology has been associated with substantial increases in revascularisation in some but not all regions. However, rates overall remain far behind those in Western Europe. Further research is in progress to understand the reasons for these variations and the barriers to further expansion of services.

## Highlights

• The substantial investment in interventional cardiology in the Russian Federation in the past 10 year has improved access to care
• Substantial geographical variations remain in terms of access to cardiology interventions
• In-hospital mortality from myocardial infarction has declined most in regions that have achieved the highest intervention rates.

## 1 Introduction

Cardiovascular disease mortality has been declining in nearly all high income countries for many decades [1]. These declines, beginning as early as the 1960s in some countries, have reflect both reduction in risk factors and improved primary and, most recently, secondary prevention [2]. Today in well-resourced settings, somebody who has a heart attack is more likely to survive than in the past, due to widely available medical interventions, including timely non-invasive percutaneous cardiovascular interventions (PCI) such as balloon angioplasty [3], [4], in which an atherosclerotic occlusion of the coronary artery is opened through inflation of a small balloon. This was introduced in the 1980s [5] and, initially, was mainly an elective procedure for people with symptomatic coronary artery disease [6], alongside coronary artery bypass grafting (CABG) which also became common at that time [7]. The extension of PCI to insert a stent, to keep the artery from closing up again, started to be introduced into routine practice in the 1990s. Today it is commonly used as the primary intervention in acute myocardial infarction. There is now good evidence that these primary reperfusion interventions result in better outcomes than thromobolysis [8] and, in many countries, there is now a switch from thrombolysis to PCI for ST-elevation myocardial infarctions (STEMI), although progress is variable [9], [10]. Yet while these minimally invasive procedures are now relatively straightforward to perform, they still require a substantial initial investment in equipment and training of expert staff.

Access to new treatments for ischemic heart disease is high on the agenda in the Russian Federation, where mortality from cardiovascular disease has been falling since 2005/6 although it remains among the highest in the world. In a speech on September 5, 2005, Russian President Vladimir Putin announced four national priority projects, including one focussed on improving population health. A council to implement the projects was established, initially headed by Putin himself and subsequently by Prime Minister Dmitry Medvedev, involving enhanced cooperation between federal, regional and local governments, non-governmental organisations, and research institutes. The health project sought to decrease the burden of disease, improve accessibility to high-quality health care, develop a prevention-oriented health care system, increase the role of primary and ambulatory care, and increase the provision of advanced medical technology. In 2008, a specific goal to reduce mortality from cardiovascular diseases was added. Increased funding was made available to improve salaries of health professionals and purchase new equipment, with federal expenditure increasing from 87.9 billion roubles in 2006–160.2 billion roubles in 2010. Initially it was planned that 80% of funds would go to primary care and disease prevention, with the 20% going to advanced medical technology. However, during the course of implementation, this shifted to a 55%/45% split. [11]

The project did much to address the long-term underinvestment in advanced medical technology in Russia. The first evaluation of the implementation of the project, in 2007, reported that 680,000 health workers had received increased salaries, 13,500 primary care physicians had undergone retraining, waiting times had reduced, and over 5500 health facilities had been re-equipped.

Its implementation has coincided with the decline in mortality from cardiovascular disease in Russia noted above. However, the role that the various federal initiatives may have played in this decline is uncertain. One study using registry data from regions acquiring new percutaneous cardiovascular intervention (PCI) centres has reported a decline in-hospital mortality [12] but otherwise there is little information on the results of the investment in advanced specialist equipment across the whole country.

In this paper, we use the available data to assess, to the extent possible, how far the investment in specialist cardiological equipment has resulted in increased numbers of PCIs (a measure of process). In addition we investigate whether at a regional level there is an association between expansion in PCI activity and changes in-hospital mortality from acute myocardial infarction among MI patients admitted to hospital in the Russian Federation.

## 2 Methods

Data on hospital activity and outcomes were obtained from the Federal Research Institute for Health Organization and Informatics of Ministry of Health of the Russian Federation. It is responsible for collating data from all Russian public hospitals, each of which is required to make regular statistical returns that are assembled initially at the regional level. These data are supplied to the Institute in the form of tabular data. Hospitals in Russia are organised in a territorial basis, at regional and federal level, with increasing levels of specialisation. In 2008, only 124 of the 6545 hospitals in Russia were privately owned and these hospitals are also required to make statistical returns. Thus, we believe that the data provide a relatively comprehensive picture of activity across the country.

We were provided with information collected on State Statistic Form 14 “Data on hospital functioning” for the years 2005–2013. This included the total number of invasive cardiovascular procedures, both Percutaneous Transluminal Coronary Angioplasty (henceforth referred to simply as PCI) and coronary artery bypass grafting (CABG). For the years 2009–2013 more detailed information was available, comprising numbers of patients with myocardial infarction (patients whose diagnosis was coded as ICD-10 I21) admitted within 12 or 24 h of symptom onset and who received thrombolysis or PCI with stenting. We present results according to two levels of geographic aggregation as defined in 2013: the 8 federal districts or the 83 constituent regional administrative regions, generally known as oblasts (Box). None of these data are broken down by age and sex.

Given the limitations of these data we adopted a pragmatic definition whereby patients deemed potentially eligible for either of these interventions were those admitted within 12 h of symptom onset, although of course this will include a variable proportion who are ineligible because of delay or contra-indications. Data on population and gross regional product of the regions of the Russian Federation were obtained from the State Statistical Committee, Rosstat.

We analysed time trends in rates of PCI per 100,000 population for the period 2005–13. We also analysed trends in the proportion of myocardial infarctions subject to PCI with stenting within 12 h of onset for the period 2009–13. We also investigated whether this progress was associated with the economic wealth of the region. Finally, we sought to determine if there was any association between greater use of primary revascularisation for myocardial infarction within 12 h of onset and in-hospital mortality. As noted, we only had data on these acute interventions from 2009, although we know that, outside a few large cities such as Moscow, it was very uncommon prior to this. Thus, it was not possible to correlate changes in acute stenting and mortality at regional level over this entire period. As a pragmatic alternative, recognising that rates were almost universally close to zero in 2005, we divided the regions into 4 categories, based on the rate of primary revascularisation in 2013 (following exclusion of those admitting fewer than 500 patients/year within 12 h of symptom onset). We then regressed in-hospital mortality by year between 2005 and 2013 in each of these regional groupings and modelled the impact on mortality from the resulting equations.

Data analyses were undertaken using SPSS. ArcGIS was used to visualise geographic variation in rates.

## 3 Results

The number and rates per 100,000 population of myocardial infarctions admitted to hospital within 24 h of onset and both PCI and coronary artery bypass grafting (not further considered) by year in the Russian Federation as a whole is shown in Table 1, for each year between 2005 and 2013.

**Table 1:** Trends in numbers and rates/100,000 population of admissions for myocardial infarction (MI) (ICD-10 I21) and selected interventions: Russian Federation.

	2005	2006	2007	2008	2009	2010	2011	2012	2013
Numbers									
MI admissions	163,301	162,58,1	161,789	161,257	162,535	155,334	152,022	152,151	156,818
PCI	12,21 8	19,426	26,296	33,872	43,129	50,390	63,412	81,416	99,664
CABG	9999	12,029	13,913	17,790	20,836	23,184	27,016	29,214	28,717
Rates/100,000									
MI admissions	116.7	116.2	115.7	115.3	116.2	111.1	108.7	108.8	112.1
PCI	8.7	13.9	18.8	24.2	30.8	36.0	45.3	58.2	71.3
CABG	7.1	8.6	9.9	12.7	14.9	16.6	19.3	20.9	20.5

*Note*: CABG − coronary artery bypass grafting.

**Fig. 1:**
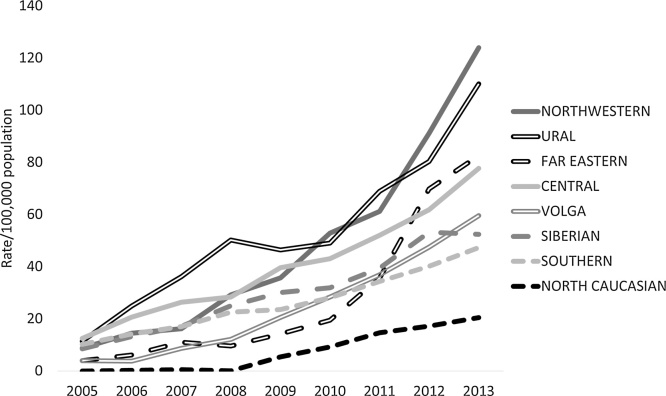
Trends in PCI/100,000 population by Russian federal district 2005–2013.

The number of PCIs has risen markedly, but at different rates per head of population across the country. Fig. 1 shows the trends in procedures in the eight federal districts. All had rates below 15/100,000 in 2005 but, by 2013, the north-western federal district, which covers the area from St Petersburg to the Urals, had achieved a rate of over 120/100,000, while in the North Caucasus it was only 20/100,000, an over a 6-fold variation.

**Fig. 2:**
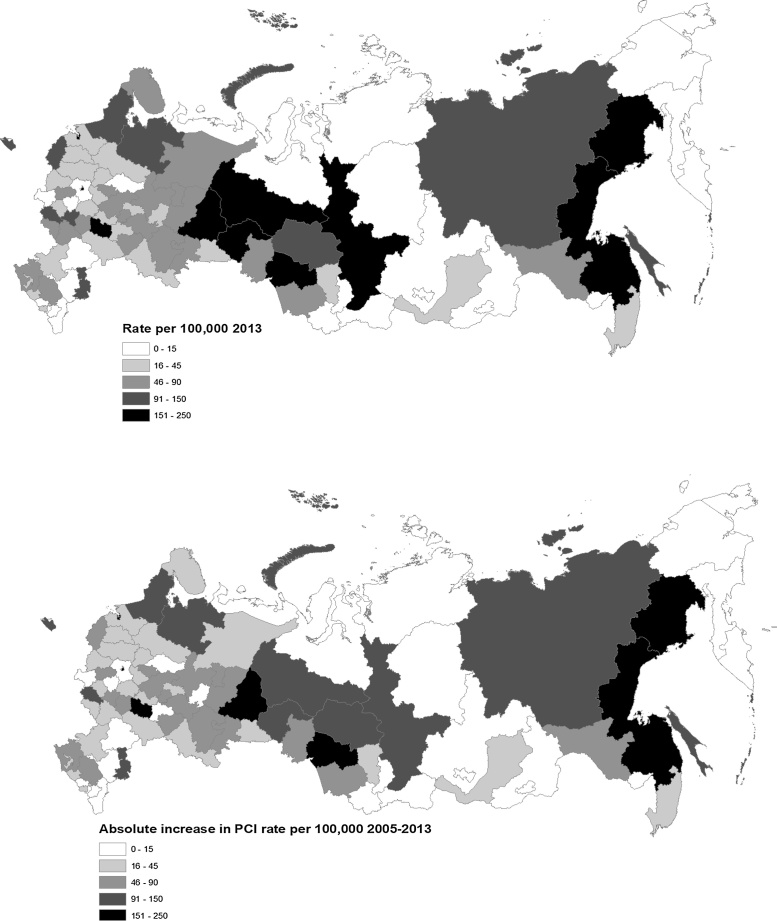
Rate of angioplasty/100,000 population in Russian regions in 2013 (top) and the absolute change between 2005 and 2013 (bottom).

A more detailed picture of geographic variation in the rate of PCI procedures is provided in Fig. 2, which maps rates at the level of regions (oblasts), divided into approximate fifths. In 2005 only five regions were undertaking more than 30 PCI per 100,000 (Moscow City, Krasnoyarsk, Tomsk, Murmansk and Tyumen). However, in 2013, 13 regions, including some of the poorest and most remote in the Far North, Far East or North Caucasus still reported no procedures. The four highest rates in 2013 were in Novosibirsk (Russia’s third-largest city and a medical and scientific research), Moscow, Magadan (in the Far East), and St Petersburg, with 241, 226, 225, and 223 procedures per 100,000 respectively.

**Fig. 3:**
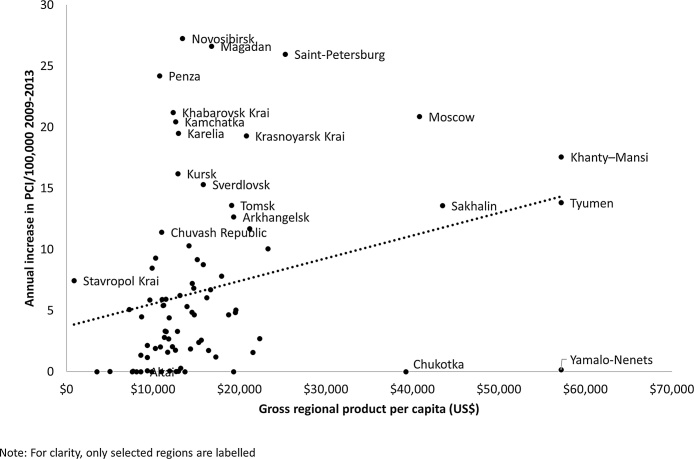
Association between annual increase in PCI/100,000 population (2005–2013) and gross regional product in Russian regions.

Intuitively, it seems likely that low population density would constrain increases in uptake. However, this is very difficult to assess with aggregate data as, in the larger regions, the population is often concentrated in a few settlements. Thus, there is no significant association between population density and the slope of the annual increase PCI rate between 2009 and 2013 (after removal of Moscow and St Petersburg that are clear outliers) (r = 0.180, p = 0.107). However, there is a significant association with wealth and the increase in uptake (Fig. 3) (r = 0.272, p = 0.013), although there are several clear outliers. The outlying wealthy regions with no or few procedures are sparsely populated mineral rich lying in the far north, such as the Yamalo-Nemets region, with a population of 500,000 in an area 20% larger than France, which produces 90% of Russia’s natural gas.

We now turn to the management and outcome of acute myocardial infarction, noting that we only have data for 2009–2013. As noted in the introduction, this has been transformed in recent decades, especially following the introduction of thrombolysis and more recently primary revascularisation using PCI. Rates of thrombolysis in hospitals have remained broadly similar during this period (Appendices), with 28.8% of patients with myocardial infarction admitted within 12 h in being given thrombolysis the Russian Federation as a whole in 2013 (from 27.9% in 2009). In contrast, there has been a steep increase in rates of primary revascularisation with stenting for which the corresponding figures were 6.5% in 2009 and 23.7% in 2013. However there is wide variation of over three-fold in 2013 between the highest and lowest performing Federal Districts and, by 2013 about two-thirds of potentially eligible patients were not undergoing revascularisation, although the data do not allow us to determine how many received thrombolysis instead. Among the individual regions, there is very wide variation (Appendices) for both treatments.

**Fig. 4:**
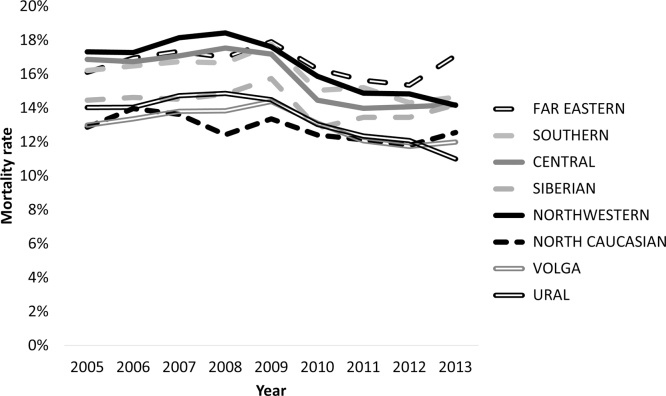
Trends in-hospital mortality following acute myocardial infarction in Russian federal districts.

Turning to outcomes, as Fig. 4 shows, there has been a decline in-hospital mortality. This coincided with a decline of 0.9% per year in the number of patients with myocardial infarction admitted to hospital over this period, consistent with the previous research cited above which noted the overall decline in cardiovascular deaths. The decline began in 2009. However, as this figure relates to in-hospital rather than over a fixed period (e.g. 30 days) some of this reduction could be due to a fall in average length of stay by patients with myocardial infarction.

The reduction in-hospital mortality was greatest in those regions achieving higher stenting rates (although the decline did not reach statistical significance in hospitals achieving stenting rates of 15–25% (Appendices).

## 4 Discussion

### 4.1 What is already known?

The high political priority placed on tackling cardiovascular disease in the Russian Federation reflects its great human, economic and social costs [13], [14]. Since the mid-2000s there has been considerable progress in reducing cardiovascular mortality. Most attention has focused on two factors that have contributed to this achievement. The first is the reduction in the high rate of alcohol-related mortality, which in post-Soviet countries includes many cardiovascular deaths, in particular sudden cardiac death [15]. This seems at least partly attributable to increased prices and measures to reduce supply [16], although deliberate alcohol control policies may not be the entire story [17]. The second is improved management of a range of conditions amenable to health care, such as diabetes and infections but also hypertension, thought to explain at least some of the decline in cardiovascular disease, especially among older women and reflecting, primarily, improved primary care and better access to medicines. However, so far, little attention has been paid to the substantial investment in advanced cardiovascular treatment, either to ascertain how it has changed treatment or whether it has improved outcomes, as might be expected given evidence from recent meta-analysis confirming improved outcomes associated with primary percutaneous intervention following acute coronary syndrome [18].

### 4.2 What this study adds

This is, to our knowledge, the first attempt in the international literature to undertake a quantitative assessment of the achievements of the Russian federal public health programme and, specifically, that element of it intended to increase access to specialised care for cardiovascular disease.

Judged by process measures, the Russian Health programme has been effective, with substantial increases in intervention rates since 2005. Revascularisation is now being undertaken in many places where it was previously absent. However there are still many regions lacking any provision. The management of acute myocardial infarction has also changed in many regions, with a marked expansion in primary revascularisation, although it remains unavailable in some regions. This mixed picture is consistent with a mid-term qualitative assessment that concluded that, while spending had increased, adding about 10% to the overall health budget, the programme had placed too much emphasis on initial capital investment without taking account of the need to train staff and fund revenue costs, including consumables [19].

It is more difficult to reach any conclusion on health outcomes. It does seem that in-hospital mortality has declined most in those regions that have achieved the highest intervention rates. However, further analysis is limited by the absence of individual level data.

There are few other data from Russia available for comparison but the international CLARIFY study, which includes 2200 patients from across Russia, reports a low frequency of revascularisation compared to other countries [20]. The RECORD study, a low cost internet based study, reported data from 14 hospitals in 2007/8 [21], with 32.1% of patients with ACS receiving thrombolysis while 18.7% received primary PCI [21]. Subsequent, so-far unpublished data from 2009 to 2011 report an increase in primary PCI to 39% but thrombolysis remaining the same, at 32%. The Russian Acute Coronary Syndrome register, operating in 23 regions [22], reported that the rate of PCI for ACS increased from 22% in 2009–2011 to 28% in 2012 [23].

Unfortunately, only a few international comparisons include Russian data, and to a limited extent [24], while others exclude Russia entirely [25]. However, comparison with published data from elsewhere illustrate the gap that remains. Comprehensive registry studies show much higher rates of primary revascularisation in Sweden (75.9%) and the United Kingdom (80.6%) in 2010 [26] although, in Europe as a whole, large variations persist, with southern and eastern Europe still achieving relatively low rates [25].

These differences, in part, may reflect the different starting points. PCI procedures in countries such as the United Kingdom have been increasing since the early 1990s, much longer than in Russia. However, there are clearly other factors; research on the increase of PCI utilization in 10 European countries has identified the role played by numbers of physicians and other health workers and of hospital beds, both of which are high in Russia. In contrast, the increase was also greatest where the population density was highest, which is not the case in most of Russia [27]. Finally, while comparisons are problematic, it is notable that rates of in-hospital mortality in Russia are appreciably higher than the 30-day mortality observed in the UK (8%) and Sweden (11%) [28].

### 4.3 Limitations

It is important to recognise the limitations of this study. Although there has been a significant investment in data collection in the Russian Federation in recent years, its systems still lag far behind those that are in place in many Western countries. Consequently, we were only able to analyse aggregate statistics at a regional level rather than data at the level of the hospital or the individual. The absence of data cross-tabulated by age is a particular limitation, especially when comparing across regions or countries. A further challenge is that, during the period that these data relate to, a number of regions have merged, in each case involving some of the largest geographical areas but with the smallest populations, most of whom are highly concentrated in a few settlements.

As with all studies using routine administrative data, considerable caution is required in interpretation. Diagnosis of myocardial infarctions would have been based on routine clinical procedures rather than on the standardised case definitions that might be used in a clinical trial, for example. However, at least for the last five years, troponin tests have been widely available.

As is the case everywhere, it is likely that there are some inaccuracies in the data. However, the returns are relatively simple, tabulating numbers of patients and procedures, and are subject to verification processes at each administrative level.

Finally, this paper only considers activity in hospitals. Especially in the more remote areas, there will be patients with myocardial infarction who do not reach hospital. Although the Russian Federation inherited a health system that provided at least basic care to a highly dispersed population, there are still many parts of the country where it can take several days to reach a hospital, simply because the distances are so vast, the climate harsh, and the transport infrastructure correspondingly limited [29]. Thus, Krasnoyarsk Krai extends from the Arctic Ocean almost to the Mongolian border. Occupying almost 1,000,000 km^2^, it has a population of 2.8 million people, a million of whom live in the regional capital in the south. On the basis of population-level data from Quebec, with a similar geography, there is likely to be substantial variation in revascularisation within these large regions [30].

## 5 Conclusion

This study shows that there has been considerable progress in improving access to high technology care in the Russian Federation coinciding with the substantial investment within the federal health programme. However, progress has been mixed and has been greatest in the major cities and lowest in the poorest regions or those which, while wealthy as judged by official statistics, have economies based on extractive industries. Unfortunately, with the data available to us at present, our ability to explain this variation is constrained. However, on the basis of our ongoing research, it is possible to make some observations. First, in some parts of the Russian Federation it is not only difficult for patients to reach hospital. It is also extremely difficult to attract and retain specialist clinical staff. It is notable that many of the regions that are still failing to provide cardiological interventions are those where the climate is extremely hostile and the population is concentrated in a few single industry settlements, based on hydrocarbon or mineral extraction. Yet, even in Moscow delays in reaching hospital are not uncommon, with a 2013 study reporting the median delay between onset of symptoms and a request for medical aid to be 2.4 h and from symptom onset to hospitalization to be 4.3 h [31]. Second, a cardiac service providing optimal care should offer primary revascularisation 24 h a day and seven days a week and, in many health facilities, patients must be transferred elsewhere for revascularisation and, even in those facilities, services are often only provided during working hours.

This paper represents a first step in undertaking the type of research on geographical variations in health care in the Russian Federation that is well established in other countries. It demonstrates the tremendous potential for undertaking natural experiments to assess the impact of health policy at a regional level. These findings are being used to inform an ongoing study that is looking in detail at the management of individual patients with myocardial infarction in 15 cities across Russia, collecting information on their health seeking behaviour and experiences prior to admission, their clinical management in-hospital, and the care that they receive in the subsequent year. This research is needed; judged by progress in reducing deaths amenable to health care, the Soviet Union failed to benefit from western medical advances during the 1970s and 1980s [32] and recent research shows the great potential for health and corresponding economic gains from improved care, such as better management of hypertension [33], [34].

Our current programme of research, while only a beginning, will provide a unique resource for understanding variation in the management of cardiovascular disease in Russia and help to build much needed capacity in health systems research in Russia.

## Competing interests

The authors declare that they have no competing interests

## References

[1] Ezzati M., Obermeyer Z., Tzoulaki I., Mayosi B.M., Elliott P., Leon D.A. (2015). Contributions of risk factors and medical care to cardiovascular mortality trends. Nature Reviews Cardiology.

[2] Yusuf S., McKee M. (2014). Documenting the global burden of cardiovascular disease: a major achievement but still a work in progress. Circulation.

[3] Degano I.R., Salomaa V., Veronesi G. (2015). Twenty-five-year trends in myocardial infarction attack and mortality rates: and case-fatality, in six European populations. Heart (British Cardiac Society).

[4] Nallamothu B.K., Normand S.L., Wang Y. (2015). Relation between door-to-balloon times and mortality after primary percutaneous coronary intervention over time: a retrospective study. Lancet (London, England).

[5] King S.B. (1998). The development of interventional cardiology. Journal of the American College of Cardiology.

[6] Ryan T.J., Faxon D.P., Gunnar R.M. (1988). Guidelines for percutaneous transluminal coronary angioplasty: a report of the American College of Cardiology/American Heart Association Task Force on Assessment of Diagnostic and Therapeutic Cardiovascular Procedures (Subcommittee on Percutaneous Transluminal Coronary Angioplasty). Circulation.

[7] Diodato M., Chedrawy E.G. (2014). Coronary artery bypass graft surgery: the past, present, and future of myocardial revascularisation. Surgery Research and Practice.

[8] Di Mario C., Dudek D., Piscione F. (2008). Immediate angioplasty versus standard therapy with rescue angioplasty after thrombolysis in the combined Abciximab REteplase Stent study in acute myocardial Infarction (CARESS-in-AMI): an open, prospective, randomised, multicentre trial. Lancet (London, England).

[9] McLenachan J.M., Gray H.H., de Belder M.A., Ludman P.F., Cunningham D., Birkhead J. (2012). Developing primary PCI as a national reperfusion strategy for patients with ST-elevation myocardial infarction: the UK experience. EuroIntervention: Journal of EuroPCR in Collaboration with the Working Group on Interventional Cardiology of the European Society of Cardiology.

[10] Kristensen S.D., Laut K.G., Kaifoszova Z., Widimsky P. (2012). Variable penetration of primary angioplasty in Europe–what determines the implementation rate?. EuroIntervention: Journal of EuroPCR in Collaboration with the Working Group on Interventional Cardiology of the European Society of Cardiology.

[11] Popovich L., Potapchik E., Shishkin S., Richardson E., Vacroux A., Mathivet B. (2011). Russian Federation Health system review. Health Systems in Transition.

[12] Boytsov S.A., Krivonos O.V., Oshchepkova E.V., Dovgalevskiy P.Y., Gridnev V.I., Dmitriev V.A. (2010). Otsenka effektivnosti realizatsii meropriyatiy, napravlennykh na snizhenie smertnosti ot sosudistykh zabolevaniy, po dannym monitoringa Minzdravsotsrazvitiya Rossii i Registra OKS [Assessing the efficiency in implementation of the measures aimed at reducing the mortality from cardiovascular disease, according to the data of Health Ministry of Russia monitoring and the ACS Register]. Menedzher zdravookhraneniya.

[13] Marquez P., Suhrcke M., McKee M., Rocco L. (2007). Adult health in the Russian Federation: more than just a health problem. Health Aff (Millwood).

[14] Kontsevaya A.V., Kalinina A.M., Koltunov I.E., Oganov R.G. (2011). Socio-economic damage by acute coronary syndrome in Russian Federation. Rational Pharmacotherapy in Cardiology.

[15] Leon D.A., Saburova L., Tomkins S. (2007). Hazardous alcohol drinking and premature mortality in Russia: a population based case-control study. Lancet (London, England).

[16] Shkolnikov V.M., Andreev E.M., McKee M., Leon D.A. (2013). Components and possible determinants of the decrease in Russian mortality in 2004–2010. Demographic Research.

[17] Grigoriev P.E.M.A. (2015). The huge reduction in adult male mortality in Belarus and Russia: is it attributable to anti-alcohol measures?. PLoS One.

[18] Huynh T., Perron S., O'Loughlin J. (2009). Comparison of primary percutaneous coronary intervention and fibrinolytic therapy in ST-segment-elevation myocardial infarction: bayesian hierarchical meta-analyses of randomized controlled trials and observational studies. Circulation.

[19] Sheiman I., Shishkin S. (2010). Russian health care: new challenges and new objectives. Problems of Economic Transition.

[20] Shal'nova S.A., Oganov R.G., Steg P.G., Ford I. (2013). Coronary artery disease in Russia: todays reality evidenced by the international CLARIFY registry. Kardiologiia.

[21] Erlikh A.D., Gratsianskii N.A. (2009). Registry of acute coronary syndromes RECORD. Characteristics of patients and results of inhospital treatment. Kardiologiia.

[22] Oshchepkova E.V., Dmitriev V.A., Gridnev V.I., Dovgalevskii P. (2012). Assessment of the quality of medical assistance for patients with acute ST elevation coronary syndrome for 2009–2010 in regions of the Russian Federation participating in the vascular program (by the data of the Russian ACS Register. Terapevticheskii arkhiv.

[23] Oshchepkova E.V., Dmitriev V.A., Gridnev V.I., Dovgalevsky P.Y. (2013). Organization of medical care of patients with ACS in the Regional Vascular Center and the primary vascular branches in 2009–2012. Eurasian Journal of Cardiology.

[24] Smith F.G., Brogan R.A., Alabas O. (2014). Comparative care and outcomes for acute coronary syndromes in Central and Eastern European Transitional countries: a review of the literature. European Heart Journal Acute Cardiovascular Care.

[25] Kristensen S.D., Laut K.G., Fajadet J. (2014). Reperfusion therapy for ST elevation acute myocardial infarction 2010/2011: current status in 37 ESC countries. European Heart Journal.

[26] Chung S.C., Sundstrom J., Gale C.P. (2015). Comparison of hospital variation in acute myocardial infarction care and outcome between Sweden and United Kingdom: population based cohort study using nationwide clinical registries. BMJ (Clinical Researched).

[27] Laut K.G., Gale C.P., Pedersen A.B., Fox K.A., Lash T.L., Kristensen S.D. (2013). Persistent geographical disparities in the use of primary percutaneous coronary intervention in 120 European regions: exploring the variation. EuroIntervention: Journal of EuroPCR in Collaboration with the Working Group on Interventional Cardiology of the European Society of Cardiology.

[28] Chung S.C., Gedeborg R., Nicolas O. (2014). Acute myocardial infarction: a comparison of short-term survival in national outcome registries in Sweden and the UK. Lancet (London, England).

[29] Levintova M. (2006). Cardiovascular disease prevention in Russia: challenges and opportunities. Public Health.

[30] Vanasse A., Niyonsenga T., Courteau J. (2005). Spatial variation in the management and outcomes of acute coronary syndrome. BMC Cardiovascular Disorders.

[31] Erlikh A.D., Matskeplishvili S.T., Gratsianskii N.A., Buziashvili Iu I. (2013). Prehospital management of patients with acute coronary syndrome in Moscow. Data of the first Moscow snapshot register. Kardiologiia.

[32] Andreev E.M., Nolte E., Shkolnikov V.M., Varavikova E., McKee M. (2003). The evolving pattern of avoidable mortality in Russia. International Journal of Epidemiology.

[33] Shum K., Alperin P., Shalnova S. (2014). Simulating the impact of improved cardiovascular risk interventions on clinical and economic outcomes in Russia. PLoS One.

[34] Mozheyko M., Eregin S., Vigdorchik A., Hughes D. (2012). A cross-sectional survey of hypertension diagnosis and treatment practices among physicians in Yaroslavl Region, Russia. Advances in Therapy.

