# Supplementary material for: How has the management of acute coronary syndrome changed in the Russian Federation during the last 10 years?
Source: Health Policy. 2017 Dec;121(12):1274–9. doi: 10.1016/j.healthpol.2017.09.018 (PMC5710997; doi:10.1016/j.healthpol.2017.09.018)
Supplement: Supplementary file 1 [file mmc1.docx]

**Supplemental materials**

Terminology used to describe Russian regions

The terminology of Russian regions is complex. They include oblasts, the most common type of region, each with a governor and locally elected legislature. They are commonly named after their administrative centres, such as Novosibirsk or Tomsk. In everyday usage “oblast” is often treated as synonymous with the English word “region”. Republics are nominally autonomous, each with its own constitution and legislature. However the federal government represents them in international affairs. They are linked to a specific ethnic minority, after whom they are named, such as Tatarstan, home of the Tatars, or Udmurtia, home of the Udmurts, although these minorities may be a minority in the republic. Krais are substantially the same as oblasts and the term is a historic one signifying that they were once considered to be frontier regions. There are also three federal cities, Moscow, St. Petersburg and Sevastapol. Finally, there are a small number of autonomous okrugs, regions linked to a particular ethnic minority but typically smaller than a republic, and in the Far East a single Jewish Autonomous Oblast, also referred to as Yevrey, derived from Yiddish, although now with only a few percent of the population who identify as Jews. During the period covered by these data the regions were grouped into eight larger Federal Districts.

Note: the 100% thrombolysis rate in Chukotka, a sparsely populated region on the Barents Sea with a largely nomadic population is based on only 6 patients who reached hospital within 12 hours.


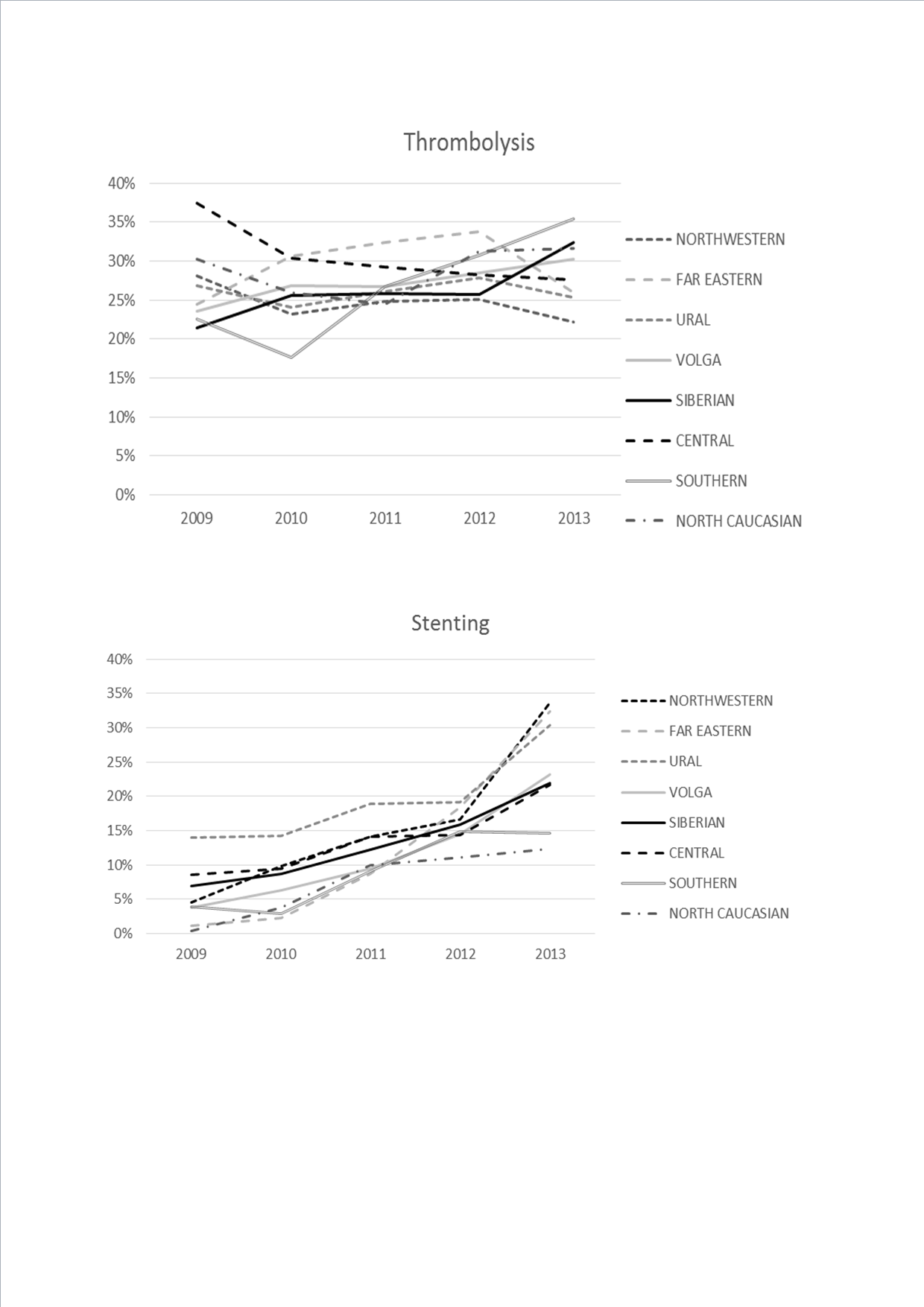


Fig. 1 Trends in the ratio of thrombolysis and primary revascularisation with stenting to patients admitted within 12 hours of the onset of a myocardial infarction in Russian federal districts

Table 1 Reduction in regional in-hospital mortality with differing rates of stenting in 2013

|  | | | | In-hospital mortality from myocardial infarction (%) with relative and absolute decline | | | |
| --- | --- | --- | --- | --- | --- | --- | --- |
| Regional stenting rate in 2013 | Annual change in in-hospital mortality (%) | 95% confidence interval | p | Morta-lity 2005 | Morta-lity 2013 | Relative decline | Absolute decline (percentage points) |
| <15% | -0.168 | -0.307;-0.030 | 0.018 | 15.6% | 14.3% | 8.3% | 1.3 |
| 15-25% | -0.152 | -0.337;0.032 | 0.105 | 14.8% | 13.6% | 8.1% | 1.2 |
| 25-34% | -0.359 | -0.660;-0.058 | 0.02 | 14.9% | 12.1% | 18.8% | 2.8 |
| >35% | -0.412 | -0.610;-0.213 | <0.001 | 15.0% | 11.7% | 22.0% | 3.3 |
