# Supplementary material for: How has the management of acute coronary syndrome changed in the Russian Federation during the last 10 years?
Source: Health Policy. 2017 Dec;121(12):1274–9. doi: 10.1016/j.healthpol.2017.09.018 (PMC5710997; doi:10.1016/j.healthpol.2017.09.018)
Supplement: Supplementary file 2 [file mmc2.docx]

**Supporting information**

S1. Appendix Terminology used to describe Russian regions

S2 Fig. 1 Percentage of myocardial infarctions admitted within 12 hours receiving thrombolysis

S3 Fig. 2 Percentage of myocardial infarctions admitted within 12 hours undergoing stenting
